# Surgical phase and instrument recognition: how to identify appropriate dataset splits

**DOI:** 10.1007/s11548-024-03063-9

**Published:** 2024-01-29

**Authors:** Georgii Kostiuchik, Lalith Sharan, Benedikt Mayer, Ivo Wolf, Bernhard Preim, Sandy Engelhardt

**Affiliations:** 1 Department of Cardiac Surgery, Heidelberg University Hospital, Heidelberg, Germany; 2 DZHK (German Centre for Cardiovascular Research), Partner Site Heidelberg/Mannheim, Heidelberg, Germany; 3 Department of Simulation and Graphics, University of Magdeburg, Magdeburg, Germany; 4 Department of Computer Science, Mannheim University of Applied Sciences, Mannheim, Germany

**Keywords:** Data visualization, Surgical workflow recognition, Surgical data science, Instrument detection

## Abstract

**Purpose:**

Machine learning approaches can only be reliably evaluated if training, validation, and test data splits are representative and not affected by the absence of classes. Surgical workflow and instrument recognition are two tasks that are complicated in this manner, because of heavy data imbalances resulting from different length of phases and their potential erratic occurrences. Furthermore, sub-properties like instrument (co-)occurrence are usually not particularly considered when defining the split.

**Methods:**

We present a publicly available data visualization tool that enables interactive exploration of dataset partitions for surgical phase and instrument recognition. The application focuses on the visualization of the occurrence of phases, phase transitions, instruments, and instrument combinations across sets. Particularly, it facilitates assessment of dataset splits, especially regarding identification of sub-optimal dataset splits.

**Results:**

We performed analysis of the datasets Cholec80, CATARACTS, CaDIS, M2CAI-workflow, and M2CAI-tool using the proposed application. We were able to uncover phase transitions, individual instruments, and combinations of surgical instruments that were not represented in one of the sets. Addressing these issues, we identify possible improvements in the splits using our tool. A user study with ten participants demonstrated that the participants were able to successfully solve a selection of data exploration tasks.

**Conclusion:**

In highly unbalanced class distributions, special care should be taken with respect to the selection of an appropriate dataset split because it can greatly influence the assessments of machine learning approaches. Our interactive tool allows for determination of better splits to improve current practices in the field. The live application is available at https://cardio-ai.github.io/endovis-ml/.

**Supplementary Information:**

The online version contains supplementary material available at 10.1007/s11548-024-03063-9.

## Introduction

Technologies that enable next-generation context-aware systems in the operating room are currently intensively researched in the domain of surgical workflow recognition [1]. Recent studies that apply machine learning algorithms to this task have shown highly promising results [2, 3]. To further support advances in this area, academic machine learning competitions are hosted regularly [4–6]. However, despite the progress in surgical workflow recognition, the developers of machine learning algorithms are faced with several challenges that result from the heterogeneous nature and complexity of surgical workflows, as well as the temporal correlation of sensor data.

Specifically, one of the major challenges of the surgical workflow data lies in the unequal distribution of classes (i. e., surgical phases) [7–15], which is commonly referred to as data imbalance in the machine learning literature [16]. This phenomenon occurs due to characteristics of surgical workflows, as individual phases and surgeries can vary significantly in their duration [17] and execution [18]. This issue is further exacerbated by the fact that some phases can re-occur several times during surgery while some phases can be optional [13, 14]. This results in an imbalanced representation of classes in the dataset which in turn hinders the ability of machine learning classifiers to accurately predict the underrepresented classes [16]. Besides, the surgical phases strongly correlate with the instruments that are used during the phase [19, 20]. Therefore, unequal distribution of phases also affects the distribution of sub-properties in the datasets, such as surgical instruments [21]. Most importantly, when splitting such datasets into training, validation, and test set, it is necessary to ensure that the dataset splits are representative and cover all classes in order to obtain reliable evaluation results [16].

In this work, we present an interactive data visualization application that facilitates the assessment of dataset splits for surgical phase and instrument recognition with regard to the aforementioned challenges. The main goal of this work is to provide a data visualization tool that can be used by machine learning practitioners as well as biomedical challenge organizers to gain insights into dataset splits of surgical workflow data.

## Related work

With the advent of deep learning, the topics of automatic phase and instrument recognition have gained considerable traction. In one of the earliest studies on this topic, Twinanda et al. [7] train a convolutional neural network for the joint phase and instrument recognition and apply a hidden Markov model to enforce temporal dependencies of phase predictions. Jin et al. [22] present an improvement upon the previous work by training a deep convolutional network and a recurrent neural network in an end-to-end manner. Furthermore, a multi-stage temporal convolutional network has been successfully applied to the task of surgical phase recognition by Czempiel et al. [11]. In the recent works, the focus has shifted toward the transformer architectures [12, 23–26].

Data visualization techniques represent a promising approach that can facilitate the exploration of surgical workflows. Yet, only limited research on visualization techniques for the analysis of surgical workflows has been conducted so far. Previously, Blum et al. [27] proposed a method based on Bayesian model merging to derive a workflow model from a set of procedures and visualize it as a graph. One of the most recent studies by Mayer et al. [28] presents an interactive visualization method that focuses on the analysis of temporal relationships within the surgical workflow data and provides means for comparing sets of procedures (e.g., stratified by surgeon, pathology, etc.).

To the best of our knowledge, only two works addressing the analysis of dataset splits for surgical phase or instrument recognition have been published so far. Fox and Schoeffmann [29] show that random sampling of video frames without considering patient split might result in training and test sets containing video frames that are visually similar. This significantly distorts the evaluation results on the test set and yields overly optimistic results. Sahu et al. [8] redefine the task of instrument detection as a multi-label recognition task in order to account for co-occurrences of surgical instruments. Due to high imbalance of the dataset, the authors perform a stratified split on instrument co-occurrences which improves the performance of the classifier in comparison with other stratification approaches. Further, the work presents methods for the quantification of dataset imbalances.

## Visualization framework

The proposed visualization framework aims to facilitate interactive exploration of dataset splits for surgical workflow recognition. In essence, this framework processes frame-wise phase and binary instrument annotation data to derive further attributes of surgeries that are crucial for creating representative dataset splits. Using the phase annotations, we sum the number of video frames that are assigned to each phase (i.e., *phase occurrence*). Furthermore, we calculate the frequency of sequential occurrence of two phases (i.e., *phase transitions*) and derive the overall duration by counting the total number of video frames of each surgery (i.e., *surgery duration*). Using the instrument annotation data, we count the number of frames each instrument is visible (i.e., *individual instrument occurrence*). However, this representation does not reflect the complexity of surgical instrument annotations since the instruments can be used simultaneously and are therefore not mutually exclusive. For this reason, we also count video frames in which two or more instruments co-occur (i.e., *instrument co-occurrence*). In the next step, these attributes are aggregated over surgeries and dataset splits. Finally, the data are presented in form of interactive visualizations. All attributes are represented by the number of video frames in which they are annotated, except for phase transitions which are described by the number of times they occur, as they do not have a temporal dimension.

The framework is implemented as a web application, using the D3 [30] library for the visualization of data. For the representation of the previously discussed attributes of surgeries, the user interface is divided into four separate views. The two main views *Phase view* and *Instrument view* specifically focus on the visualization of surgical phases and instruments, respectively. Two further supplementary views provide an overview of the assignment of surgeries to the dataset splits as well as their durations. The colors red, green, and blue are used consistently across all views to encode attributes of the training, validation, and test set, respectively. All of the four views are interlinked, thus allowing to explore the correspondences between various attributes by filtering the data based on an attribute in one view and inspecting the filtered data in the adjacent view. The following sub-sections introduce individual views of the user interface.

### Phase view

**Fig. 1:**
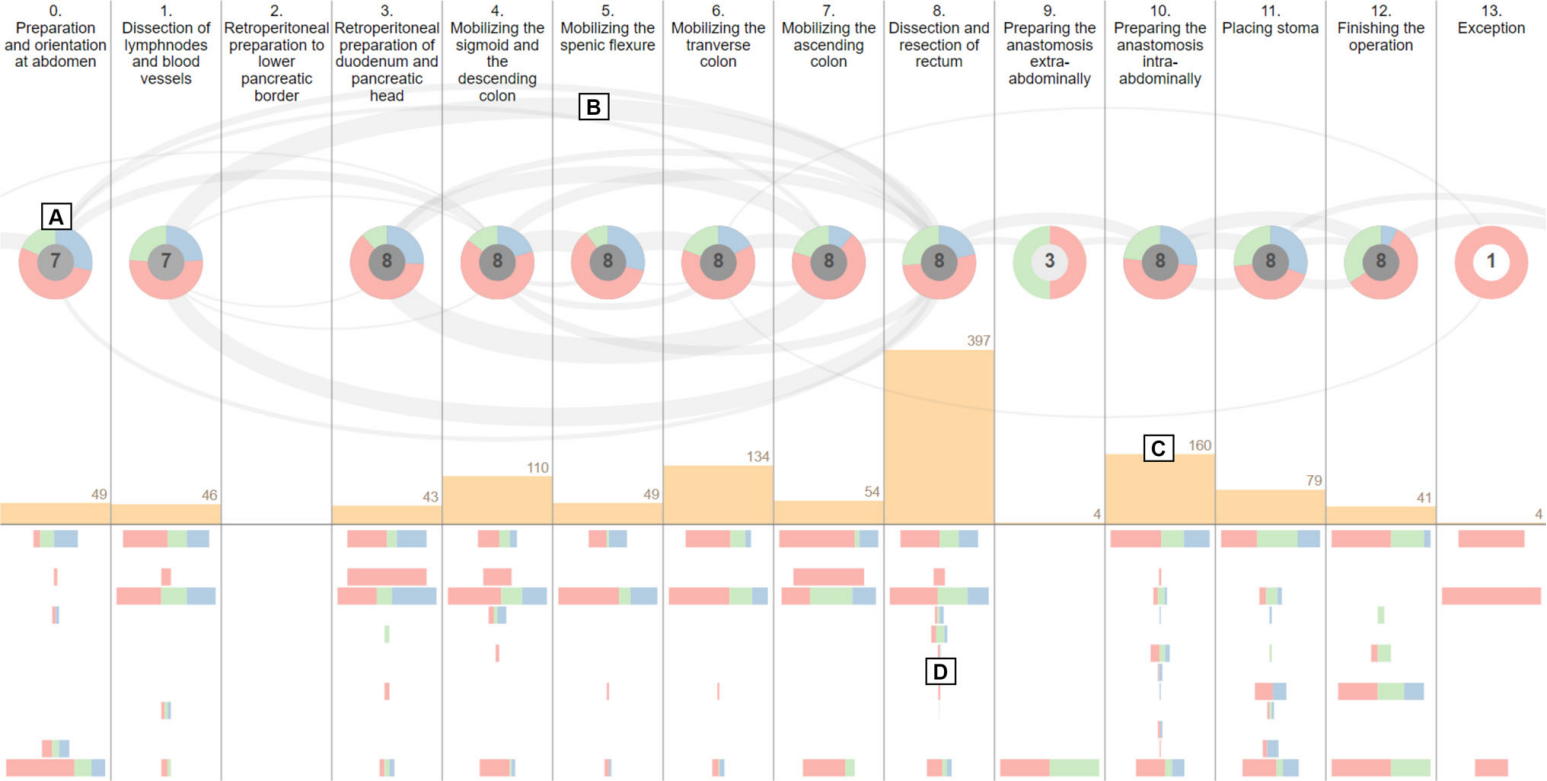
Phase view of the proposed application with eight proctocolectomy surgeries from the “Surgical Workflow Analysis in the sensorOR 2017” challenge dataset [6]. The nodes show the phase occurrence across splits and surgeries (**A**). Transitions between phases are visualized as arcs (**B**). The bar chart in the middle of the view shows the total number of frames per phase (**C**). The centered vertical bar charts at the bottom display the occurrence of individual instruments per phase (**D**)

In this view, phase occurrences are visualized as nodes along the horizontal axis, ordered according to their conceptual order from left to right (see Fig. 1A). The visualization is based on the Arc Diagram visualization method [31]. Each node contains a donut chart that represents the proportion of frames that are assigned to the corresponding dataset split. The colors red, green, and blue encode the attributes of the training, validation, and test set, respectively. Furthermore, the center of each node shows the number of surgeries in which the phase occurs. Phase transitions are visualized as arcs between individual nodes, whereas the number of times a transition between two phases happened is mapped to the width of arcs (see Fig. 1B). Since transitions can occur in both directions, forward transitions are displayed in the upper half, while backward transitions are placed in the lower half of the chart. The transitions starting from the left side of the view or ending in right side represent start and end of the surgeries. The overall distribution of frames across surgical phases is displayed as a bar chart below the phase nodes (see Fig. 1C). Finally, the horizontal bar charts at the bottom of the view show individual instrument occurrence per each phase (see Fig. 1D).

In order to support interactive exploration of the data, several interaction techniques are implemented in the phase view. By selecting individual phase nodes, filtering is applied across other views to display frames for the selected set of phases. Furthermore, surgeries can be filtered by the occurrence of a particular phase transition. The *Phase view* and other views are updated accordingly to display the surgeries that contain the selected transition. Besides, the occurrence of phase transitions in the training, validation, and test sets can be displayed as pie charts placed over each transition arc upon selecting the corresponding option in the *phase view* menu.

### Instrument view

**Fig. 2:**
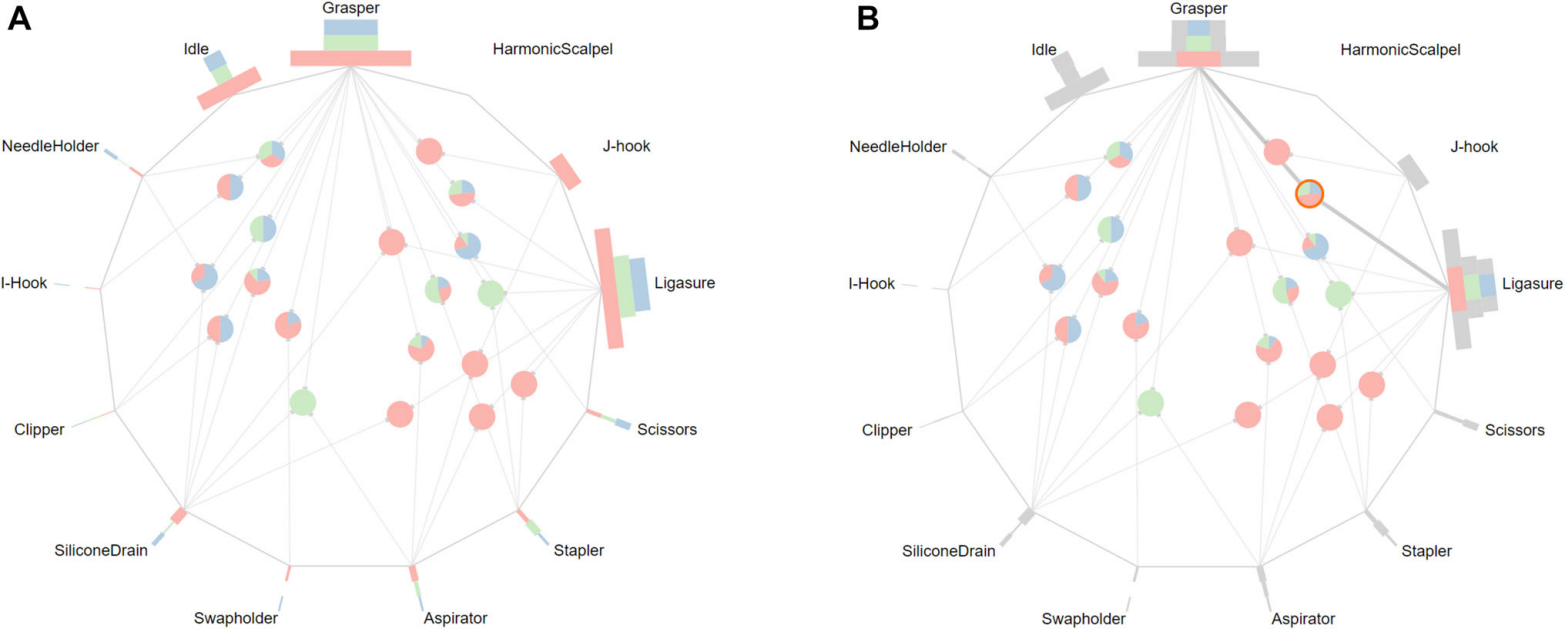
Instrument view of the proposed application with eight proctocolectomy surgeries from the “Surgical Workflow Analysis in the sensorOR 2017” challenge dataset [6] (**A**) and selected combination of *Grasper* and *Ligasure* (**B**)

The *instrument view* addresses the visualization of individual instrument occurrences as well as the instrument co-occurrences (see Fig. 2A). The colors red, green, and blue encode the attributes of the training, validation, and test set, respectively. This visualization approach is based on the Radial Sets technique by Alsallakh et al. [32] which targets the analysis of set memberships of data elements. The centered bar charts which are arranged radially show the total number of frames a surgical instrument was visible in each set (i.e., individual instrument occurrence). Additionally, a bar chart that reflects the number of frames in which no instruments are visible, so-called idle frames, is also included in this view. The combinations of instruments (i.e., instrument co-occurrences) are displayed as nodes in the center of the *instrument view*. The nodes themselves are represented as pie charts, whereas each segment of the pie chart shows the prevalence of this instrument combination in the training, validation, and test set. The positioning of the nodes is determined by a force-directed layout algorithm implementation of the D3 library [30].

To facilitate the exploration of the surgical instrument data, several interaction techniques are implemented in this view. By selecting an individual instrument, all instrument co-occurrence nodes that involve the selected instrument are highlighted in the *Instrument view*. Besides, co-occurrence nodes can be selected individually which reveals the proportion of co-occurrence frames in relation to the frames of the involved instruments (see Fig. 2B). Upon filtering of individual instruments or instrument co-occurrences, other views of the visual framework are updated accordingly to view the selected frames.

### Supplementary views

**Fig. 3:**
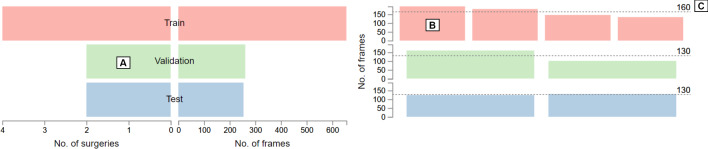
Supplementary view of the proposed application. Two mirrored bar chars show the number of surgeries and the total number of video frames in the training, validation, and test set (**A**). A set of three bar charts display the duration (i.e., number of frames) of each surgery (**B**). The dashed lines show the average surgery duration per set (**C**)

The main views are enhanced by two supplementary views which provide a general overview of the dataset. The colors red, green, and blue encode the attributes of the training, validation, and test set, respectively. The first supplementary view represents a table that shows the partitioning of surgeries into the training, validation, and test sets. The individual surgeries can be interactively re-assigned to a different set via drag and drop. The second supplementary view encompasses two bar charts that display the total number of surgeries and frames for each set (see Fig. 3A). Additionally, a set of bar charts displaying the number of frames for each individual surgery are arranged on the right side of the view (see Fig. 3B). The average number of frames for each set is shown as dashed lines in the bar charts (see Fig. 3C).

## Evaluation and results

The proposed visualization framework is evaluated through a user study using the Cholec80 dataset [7]. In addition to the user study, we use our framework to analyze splits of five popular datasets for the surgical phase and instrument recognition tasks, highlight problematic cases, and propose optimized splits.

### User study

In total, ten participants with data science background have been recruited to participate in the evaluation study of the proposed visualization framework. After a brief introduction into the domain of surgical phase recognition and the features of the proposed application, the participants were asked to solve ten tasks covering a wide range of possible exploratory analyses that can arise during the preparation of Cholec80 dataset [7]. Further details on the user study are provided in the supplementary information. To measure the results of this study, task completion percentage was used, which has the value of 1 only if the participant solves the task correctly, 0 otherwise. Overall, the majority of the tasks were completed successfully by $\ge 80\%$ of participants.

After completing the tasks, the participants were asked to fill out the System Usability Scale (SUS) [33] questionnaire. It consists of ten statements that the study participants ranked on a 5-point Likert scale ranging from 1 (strongly disagree) to 5 (strongly agree). The ranking of the statements is then used to calculate the SUS score which expresses the usability of the system. The value of the score ranges between 0 and 100, with higher values expressing better usability. The proposed application reached the SUS score of 81.25.

### Analysis of dataset splits

In order to validate the proposed framework, we perform analysis of various dataset splits for the Cholec80 [7], CATARACTS [10], CaDIS [34], as well as the M2CAI workflow and tool datasets [7, 35] using our visualization framework, report our observations, and propose improvements in the dataset splits. The datasets represent a diverse selection of surgical procedures, workflows, surgical instruments, dataset splits, as well as annotation and data types.

#### Analysis of the Cholec80 dataset

For the analysis of the Cholec80 dataset splits, we chose the three most common Cholec80 splits [15]. We downsampled phase annotations of the Cholec80 dataset to 1 fps to obtain frames with both phase and instrument labels.

***40/-/40 split***

**Fig. 4:**
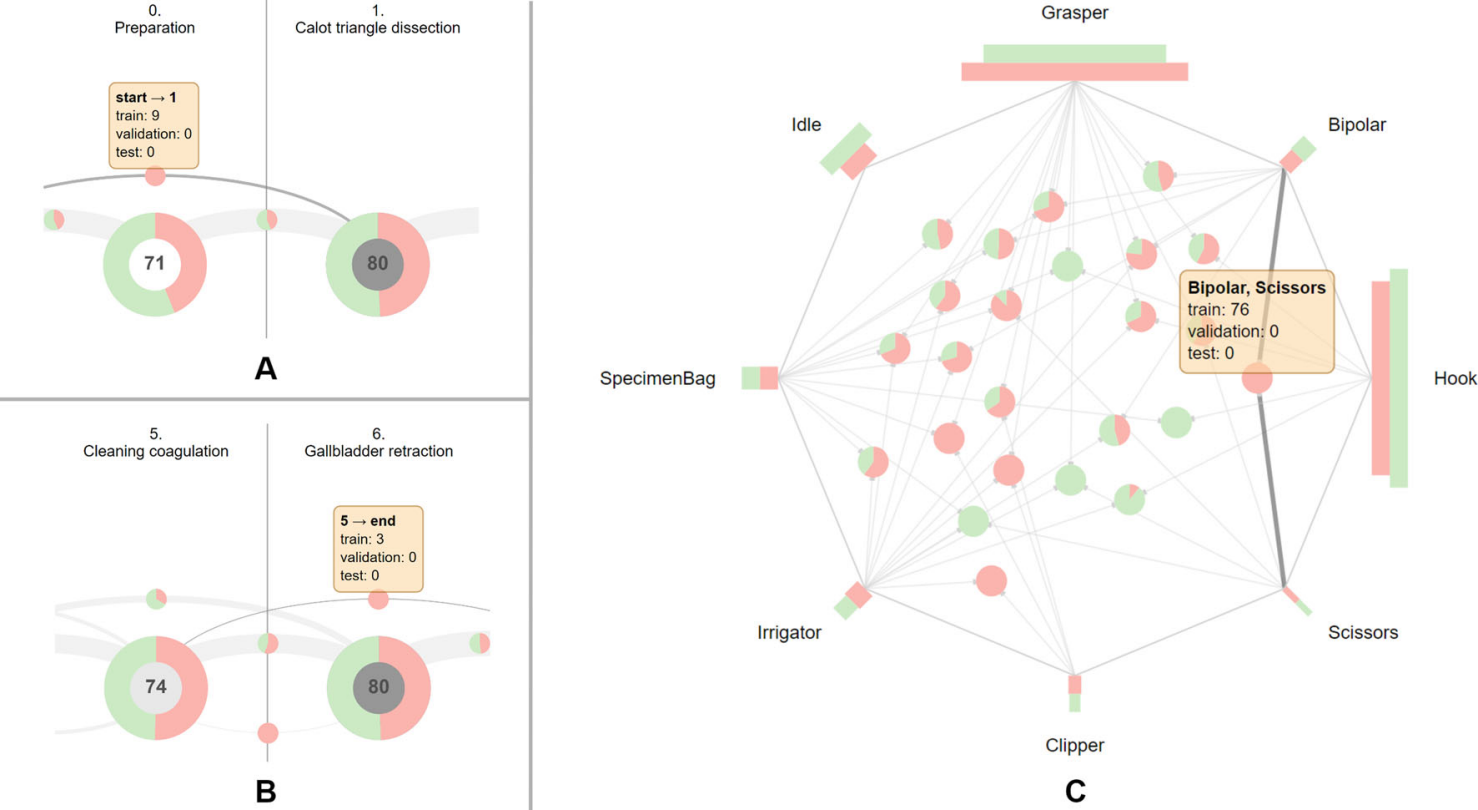
Characteristics and shortcomings of the 40/-/40 split of the Cholec80 dataset [7]. Surgeries starting in the *Calot triangle dissection* phase are only present in the training set (**A**). The ending sequence *Gallbladder retraction* to *Cleaning coagulation* occurs only in the training set (**B**). The instruments *Bipolar* and *Scissors* co-occur only in the training set (**C**)

In the 40/-/40 split, which is used in the studies [7, 36], all surgical phases are represented in both sets. However, a closer inspection of phase transitions unveils a group of nine surgeries (10, 13, 19, 22, 23, 29, 32, 33, 38) that deviate from the standard workflow by skipping the first phase and initiating the surgery directly in the second phase (see Fig. 4A). Notably, all of the nine surgeries are assigned to the training set; therefore, the evaluation of the model’s performance on the test set does not include this special workflow. In addition, another unique workflow that only occurs in three surgeries (12, 14, 32) in the training set can be identified using the proposed visualization (see Fig. 4B). After the *Gallbladder packaging* phase, these three surgeries move on to the *Gallbladder retraction*, thus omitting the *Cleaning coagulation* phase. Subsequently, the surgeries return to the previously skipped *Cleaning coagulation* phase which is also the final phase of the three surgeries. Since this unique sequence of phases only appears in the training set, they are not included in the evaluation of the machine learning model. **Proposed improvement:** With this information at hand, the split can be optimized by re-assigning the surgeries 29, 32, 33, and 38 to the test set, as interactively determined in our tool. Accordingly, four randomly selected surgeries 58, 66, 71, 78 from the test set are assigned to the training set to retain the 40/-/40 split. As a result of this re-partition, the aforementioned cases of phase transitions now also appear in the test set.

Regarding the instrument use, the proposed visualization shows that all of the individual instruments are represented in all sets and also follow similar distributions. Nevertheless, there are several instrument combinations that do not occur in one of the sets (see Fig. 4C). However, these instruments combinations mostly represent rare cases, as they account for only a small fraction of the dataset and appear in single surgeries.

***32/8/40 split***

**Fig. 5:**
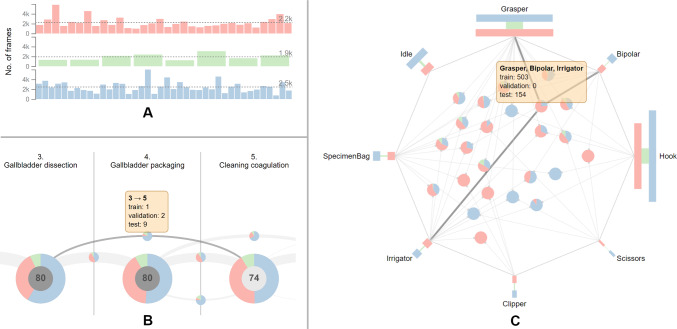
Characteristics and shortcomings of the 32/8/40 split of the Cholec80 dataset [7]. Surgeries from the validation set have fewer frames on average, compared to the training and test sets (**A**). The phase transitions (*Gallbladder dissection*, *Cleaning coagulation*) and (*Cleaning coagulation*, *Gallbladder packaging*) occur only once in the training set (**B**). The simultaneous occurrence of the instruments *Grasper*, *Bipolar*, and *Irrigator* is not represented in the validation set (**C**)

To perform model selection or hyperparameter search, studies [11, 25, 37] use eight surgeries from the training set for validation, resulting in a 32/8/40 split [15]. This split yields sufficient representation of phases across sets. However, surgeries from the validation set have fewer frames on average ($\approx$ 1900 frames) than the training and test sets with $\approx$ 2200 and $\approx$ 2500 frames, respectively (see Fig. 5A). Especially, the disparity between the average duration of surgeries from the validation and test set ($\approx 10$ min) might affect the performance estimation on these sets. As the surgery duration can indicate its complexity, the surgeries from the validation set may be easier to infer.

Similar to the 40/-/40 split, the surgeries skipping the first phase are found exclusively in the training and validation sets. Besides, the 32/8/40 split entails reduction in the training set size. This becomes especially apparent in case of two phase transitions (*Gallbladder dissection*, *Cleaning coagulation*) and (*Cleaning coagulation*, *Gallbladder packaging*) as they are reduced from three occurrences to just a single occurrence in the training set, as opposed to two and nine occurrences in the validation and test set, respectively (see Fig. 5B). This will presumably hinder the generalization of the model. **Proposed improvement:** This can be solved with our tool by re-assigning the surgery 14 to the validation set, surgeries 23, 29, 32 to the test set, and surgeries 37, 41, 57, 60 to the training set. Regarding the instruments, the co-occurrences of surgical instruments that are missing in one of the sets are more prevalent in this split due to the additional validation set. One considerable example is the simultaneous use of *Grasper*, *Bipolar*, and *Irrigator* occurring in 503 frames in the training set and in 154 frames in the test set (see Fig. 5C).

***40/8/32 split***

Instead of setting aside eight surgeries from the training set, some studies [11, 38] select eight surgeries from the testing set for validation, thus creating a 40/8/32 split. In this split, all phases as well as single instruments are present in all sets and also follow similar distributions. Similar to the original 40/-/40 split, surgeries starting in the *Calot triangle dissection* phase are exclusive to the training set. Furthermore, the three surgeries that move on from *Gallbladder packaging* to *Gallbladder retraction* and end in the *Cleaning coagulation* phase are also found only in the training set. **Proposed improvement:** This particular issue can be addressed by moving the surgeries 14, 33, 38, 57 to the validation set, the surgeries 23, 29, 32 to the test set, and the surgeries 43, 46, 47, 48, 60, 70 to the training set to retain the 40/8/32 split.

**Fig. 6:**
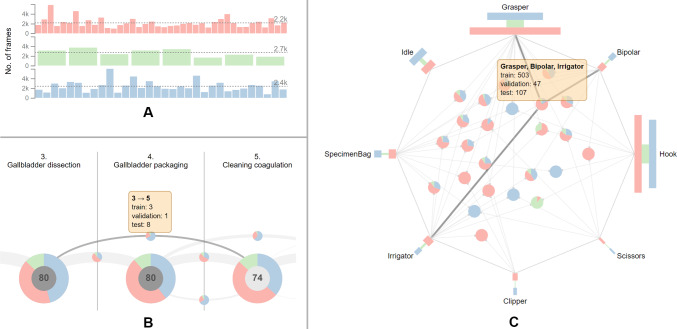
Characteristics of the 40/8/32 split of the Cholec80 dataset [7]. Surgeries from the validation set contain more frames on average than surgeries from other sets (**A**). Furthermore, this split provides a better coverage of the phase transitions (*Gallbladder dissection*, *Cleaning coagulation*) and (*Cleaning coagulation*, *Gallbladder packaging*) in the training set, compared to the 32/8/40 split (**B**). The combination of *Grasper*, *Bipolar*, and *Irrigator* appears in all sets (**C**)

Compared to the 32/8/40 split, the validation set holds a larger amount of frames, thus resulting in a better coverage of various cases (see Fig. 6A). Furthermore, the phase transitions (*Gallbladder dissection*, *Cleaning coagulation*) and (*Cleaning coagulation*, *Gallbladder packaging*) now appear three times in the training set, thus providing more samples for the training of the model (see Fig. 6B). Considering the co-occurrence of instruments, an improvement over the 32/8/40 split can be observed, as the combination of *Grasper*, *Bipolar*, and *Irrigator* now also appears on 47 frames in the validation set (see Fig. 6C).

#### Analysis of the CATARACTS dataset

The CATARACTS dataset [10] provides annotations of steps which describe the surgical procedures at a more fine-grained level compared to surgical phases. Since each step of the CATARACTS dataset is preceded by an *Idle* step, we exclude this step from the analysis to obtain a linear workflow. In the following, we inspect the suggested 25/5/20 split [10].

The inspection of the visualizations reveals that all steps are present in the training, validation, and test set. Particularly, even steps that are rare and appear only in 3 out of 50 surgeries are included in all dataset splits. Phase transitions that appear frequently prominently stand out in the visualizations. However, upon closer inspection, numerous rare transitions that are exclusive to single surgeries can be also identified. Furthermore, most of the surgeries start in the *Incision* step while two surgeries, one from the training and one from the test set, start in the *Toric Marking* step and consequently proceed to the *Incision* step.

**Fig. 7:**
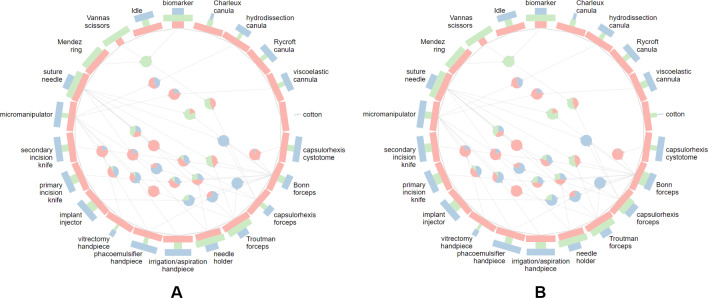
Individual instrument occurrence and the co-occurrences of the CATARACTS dataset [10] (**A**). *Mendez ring*, *Vannas scissors*, and *Cotton* are not represented in one of the sets. Individual instrument occurrence and the co-occurrences after the suggested re-partitioning to ensure that *Cotton* also appears in the validation set (**B**). The widths of the radial bar charts are scaled per each individual instrument for better visibility

Reviewing the occurrence of surgical instruments, it becomes apparent that the instruments *Mendez ring* and *Vannas scissors* generally do not appear in the test set (see Fig. 7A). Furthermore, *Cotton* is not used in the validation set and only rarely appears in the test set (see Fig. 7A). **Proposed improvement:** To achieve a better representation of *Cotton* across sets, we interactively re-assign the surgery 35 from the training to the validation set and surgery 14 from the validation to the training set. By performing these actions, we ensure that *Cotton* is also represented in the validation set (see Fig. 7B).

#### Analysis of the CaDIS dataset

The CaDIS dataset [34] consists of 25 surgeries from the training partition of the CATARACTS dataset [10] that have been annotated with the segmentation masks of anatomical structures and surgical instruments. We convert the segmentation masks of surgical instruments from the *Task III* of the original publication [34] into binary frame-wise annotation format that is required by the visualization application. Furthermore, we follow the suggested dataset split that has been specifically designed such that all instrument classes are similarly distributed across dataset splits.

**Fig. 8:**
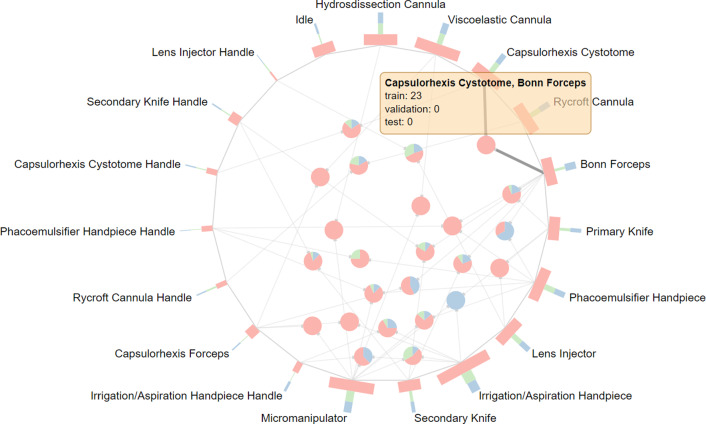
Visualization of individual instrument occurrence and the co-occurrences of the CaDIS dataset [34]. The combination of instruments *Capsulorhexis Cystotome* and *Bonn Forceps* appears exclusively in the training set

The application reveals that all individual instrument parts are indeed present in all dataset splits (see Fig. 8). Nevertheless, when examining the co-occurrences of instruments, several instrument combinations that are unrepresented in one of the sets can be identified. Particularly, several instrument combinations are exclusive to the training set. For instance, the combination of *Capsulorhexis Cystotome* and *Bonn Forceps* only appears in two surgeries with the IDs 19 and 20 from the training dataset. **Proposed improvement:** To reduce the number of unrepresented co-occurrences, the surgery 19 should be moved to the validation set, surgery 21 to the test set, and surgeries 7 and 2 to the training set. Other instrument combination from the training set are unique to individual surgeries; therefore, this issue cannot be mitigated by a re-partition on a surgery basis.

#### Analysis of the M2CAI-workflow dataset

This dataset has been introduced as part of the M2CAI EndoVis challenge 2016 and provides surgical phase annotation for a total of 41 cholecystectomy surgeries [7, 35]. For the analysis of the dataset, we downsample the annotations to 1 fps and use the dataset split that has been used in the challenge.

**Fig. 9:**
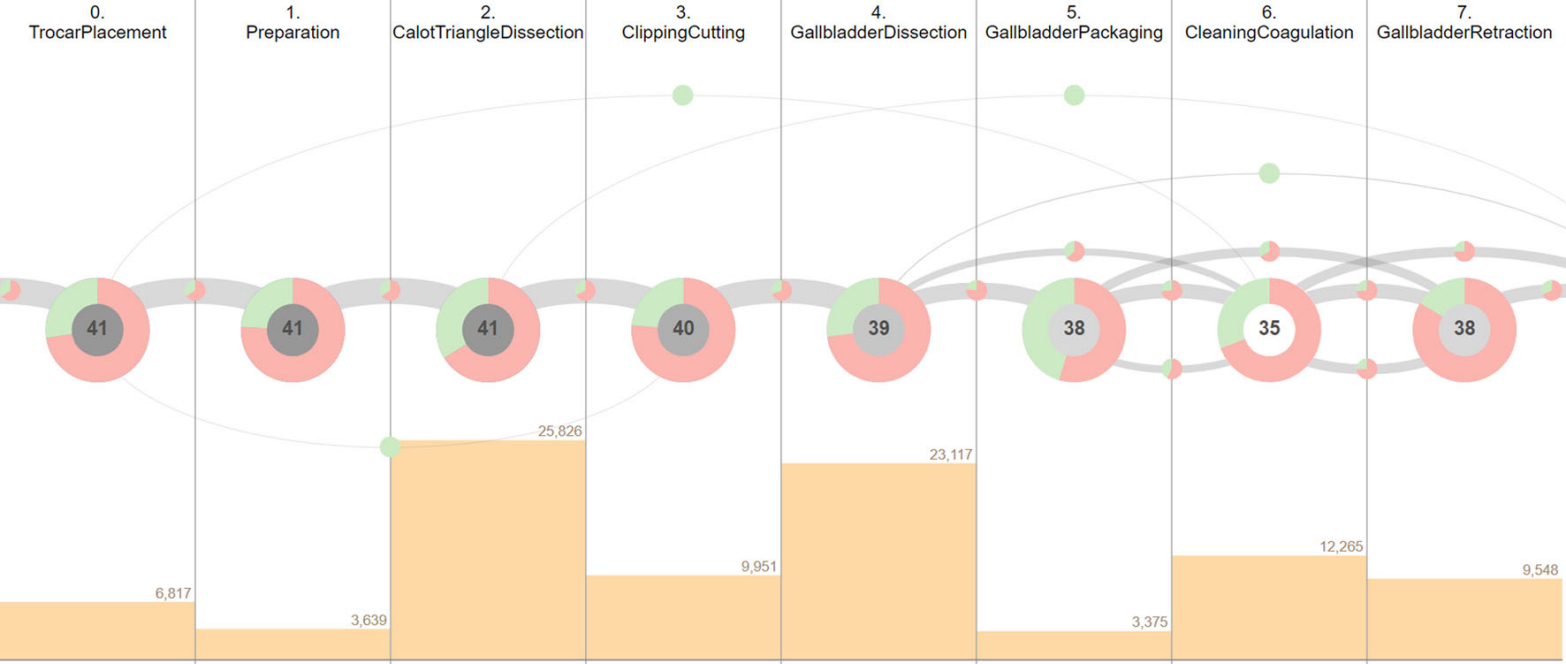
Visualization of phase occurrences and transitions from the M2CAI-workflow dataset [7, 35]

The visualizations reveal that all eight phases are represented across splits (see Fig. 9). Besides, the majority of the phase transitions occur in both training and test sets. Nevertheless, the visualization also uncovers four phase transitions that are rare and appear exclusively in the test set. These four transitions are particularly conspicuous as they skip multiple sequential phases and therefore might indicate aberrant surgical courses. Upon filtering of surgeries that contain the aforementioned transitions, it becomes evident that these surgeries generally follow unique workflows. The surgery 3 initially follows a linear workflow, starting from the first phase *Trocar placement*, consequently moving on to the *Preparation*, and then, it abruptly ends after the third phase *Calot triangle dissection* skipping five succeeding phases. Similarly, the surgeries 1 and 11 from the test set adhere to the conceptual order of the phases for the first five phases and then finish in the *Gallbladder dissection*, thus omitting the phases *Gallbladder packaging*. *Cleaning coagulation*, and *Gallbladder retraction*. **Proposed improvement:** By moving the surgery 11 from the test set to the training set and a randomly selected surgery 10 from the training set to the test set, this workflow is now represented in both training and test sets.

Furthermore, the proposed application shows that procedures from the test set are on average nine minutes shorter than the training counterpart. If the duration of the procedure indicates its overall complexity, it can be assumed that the evaluation on this test set might yield overly optimistic results.

#### Analysis of the M2CAI-tool dataset

**Fig. 10:**
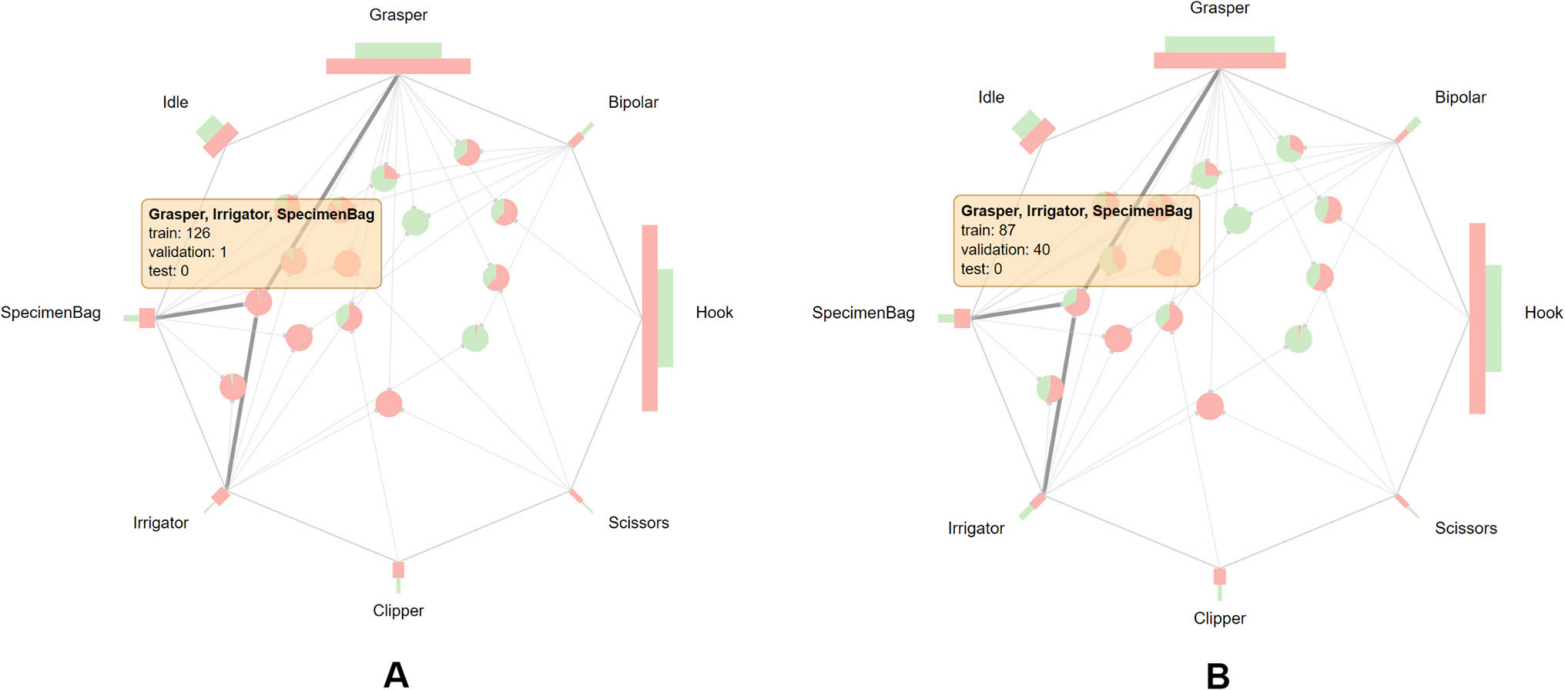
Visualization of the instrument usage of the M2CAI-tool dataset [7, 35]. Several instrument co-occurrences, e.g., *Grasper*, *Irrigator*, and *Specimen bag*, are not well distributed across the training and test, appearing only on one frame in the test set (**A**). By swapping two surgeries, these co-occurrences show an improved distribution across sets (**B**)

The M2CAI-tool dataset [7, 35] has been introduced as part of the M2CAI EndoVis challenge 2016 and provides binary instrument annotations of 15 surgeries. For the analysis of the dataset, we follow the suggested split of 10/-/5 [35]. The visualizations show that all individual instruments are included in the training and test sets (see Fig. 10A). With respect to the instrument combinations, there are four combinations that appear exclusively in one of the sets and are unique to a single surgeries. Further three combinations are heavily imbalanced, for instance, the combination of *Grasper*, *Irrigator*, and *Specimen bag* with 126 frames in training set and a single frame in the test, or the combination of *Bipolar* and *Irrigator* with a single frame in the training set and 28 frames in the test set. **Proposed improvement:** By switching the surgeries 6 and 14, the distribution of instrument combinations across dataset splits can be significantly improved (see Fig. 10B). The combination of *Grasper*, *Irrigator*, and *Specimen bag* is now split into 87 and 40 frames in the training and test set, respectively.

### Summary of unrepresented cases

Table 1 shows dataset splits of the five datasets as well as the number of phase transitions, and instrument combinations that are not represented in one of the sets. The improved dataset splits that are presented as part of this work are denoted with *.

**Table 1:** Number of phase transitions, instrument co-occurrences, and individual instruments that are unrepresented in one of the sets and were discovered using the proposed visualization framework. Improved splits proposed as part of this work are indicated with *

Dataset	Split^a^	Publications	Unrepresented attributes								
			Phase transitions			Instrument co-occurrences			Individual instruments		
			Train	Val	Test	Train	Val	Test	Train	Val	Test
Cholec80	40/-/40	[7, 36]	0	–	3	4	–	4	0	–	0
	40/-/40*		0	–	0	4	–	4	0	–	0
	32/8/40	[22, 25, 37]	0	2	3	4	14	4	0	0	0
	32/8/40*		0	0	0	6	11	3	0	0	0
	40/8/32	[11, 38]	0	3	3	4	9	6	0	0	0
	40/8/32*		0	0	0	6	10	3	0	0	0
CATARACTS	25/5/20	[10]	30	36	33	4	11	6	0	1	2
	25/5/20*		30	35	33	4	11	6	0	0	2
CaDIS	19/3/3	[34]	–	–	–	1	12	9	0	0	0
	19/3/3*		–	–	–	1	9	9	0	0	0
M2CAI-workflow	27/-/14	[7, 22, 23, 35]	4	–	0	–	–	–	–	–	–
	27/-/14*		3	–	0	–	–	–	–	–	–
M2CAI-tool	10/-/5	[7, 8, 35]	–	–	–	1	–	3	0	–	0
	10/-/5*		–	–	–	1	–	3	0	–	0

^a^Number of surgeries assigned to the training/validation/test sets

## Discussion and future work

This work presents a publicly available visualization framework that facilitates interactive assessment of dataset splits for surgical phase and instrument recognition. The motivation for this has been previously outlined in some studies. Zisimopoulos et al. [9] report a high discrepancy of the model’s performance on validation and test sets which is attributed to some phases missing in the validation set. The problem of the inherent data imbalance of surgical workflow data has been previously highlighted in several works [7–15]. The visualization framework presented in this work is specifically designed to address these cases.

To validate the design of our application, we analyzed five common datasets using our tool. We were able to pinpoint several aspects of the dataset splits that can distort the evaluation of the model’s performance. Moreover, the application enabled us to eliminate some of these issues by interactively re-partitioning the sets. Nevertheless, the proposed visualization also bears certain limitations. The visualization of phase transitions solely shows the frequency each individual phase transition occurs in the dataset. While this visualization approach allows to successfully identify phase transitions that are unique, determining whether a particular sequence of transitions appears in a surgery can only be achieved by applying filtering in the *Phase view*. Therefore, unique workflow patterns may remain undiscovered by using the proposed application. The previous work by Blum et al. [27] presents a more suitable approach for the analysis of workflow patterns. Further, the visualization provides a heavily aggregated view of surgical phases and does not provide a visual representation of re-occurrences of phases, in case a phase has been repeated multiple times during a surgery. The work by Mayer et al. [28] allows for the understanding of the temporal relationships within surgical workflow data.

While the visualization of instruments displays total number of video frames per each individual instrument as well as the frames in which two or more instruments co-occur, it does not provide a clear visual representation of video frames in which only a single instrument is used. To view such cases, the user is required to perform filtering in the *Instrument view*, consequently making them less apparent. This issue should be addressed in the future work in order to provide a complete overview of the instrument usage data.

Using the insights from our visualization tool, we were able to successfully re-partition the datasets to achieve a better distribution of attributes across dataset splits. However, the re-partitioning was performed manually and likely does not represent the most optimal splitting. In future work, algorithms for the generation of optimal dataset splits [39] can be explored. Besides that, our analysis of dataset splits and the recommendations derived from it need to be supported by quantitative evaluations in the future work.

Further, the scope of this application is limited to the analysis of phase and instrument annotations. However, visual features, such as bad lighting conditions, over or underexposed instruments, and occlusions, have high influence on the performance of the model [22] and should be considered in the future work. Correspondingly, it can be also extended to support adjacent tasks including instrument and pathology detection or segmentation with bounding-box or pixel-level predictions to account for spatial relationships of the data. Finally, we also believe that integration of more fine-grained surgical activity information, such as action triplets [40], can provide a more sophisticated overview of surgical workflows.

## Conclusion

In this work, we presented a publicly available application implemented for the research community that aims to facilitate visual exploration of dataset splits for surgical phase and instrument recognition. To validate the design of our application, we conducted a user study with ten participants. Further, we performed an analysis of common surgical phase and instrument recognition datasets and identified improvements in the splits using our tool. The results indicate that the proposed application can enhance the development process of machine learning models for surgical phase recognition by providing insights into the dataset splits, potentially resulting in more reliable performance evaluations. Furthermore, we believe that organizers of biomedical challenges can also greatly benefit from the proposed framework during the preparation of challenge datasets.

### Supplementary Information

Below is the link to the electronic supplementary material.

Supplementary file 1 (pdf 190 KB)

## Data Availability

Source code is available at https://github.com/Cardio-AI/endovis-ml and the live application can be accessed at https://cardio-ai.github.io/endovis-ml/.
