# Supplementary material for: Surgical phase and instrument recognition: how to identify appropriate dataset splits
Source: Int J Comput Assist Radiol Surg. 2024 Jan 29;19(4):699–711. doi: 10.1007/s11548-024-03063-9 (PMC10973055; doi:10.1007/s11548-024-03063-9)
Supplement: Supplementary file 1 — (pdf 190 KB) [file 11548_2024_3063_MOESM1_ESM.pdf]

Surgical Phase and Instrument Recognition: How to identify appropriate Dataset Splits (Supplementary Information)

Georgii Kostiuchik[1,2*], Lalith Sharan[1,2], Benedikt Mayer[3], Ivo Wolf[4], Bernhard Preim[3] and Sandy Engelhardt[1,2]

[1]Department of Cardiac Surgery, Heidelberg University Hospital, Heidelberg, Germany.
[2]DZHK (German Centre for Cardiovascular Research), partner site Heidelberg/Mannheim, Germany.
[3]Department of Simulation and Graphics, University of Magdeburg, Magdeburg, Germany.
[4]Department of Computer Science, Mannheim University of Applied Sciences, Mannheim, Germany.

*Corresponding author(s). E-mail(s): georgii.kostiuchik@med.uni-heidelberg.de;

1 User study

The following Table 1 shows tasks from the user study of the presented visualization framework.

Table 1 List of tasks to be solved by study participants using the visualization framework.

ID	Description
T1	Which phase has the most frames?
T2	Which two phases have the largest proportion of idle frames?
T3	Which instruments co-occur with the Clipper?
T4	Which phase is not represented in all dataset splits?
T5	Which phases are not present in all surgeries?
T6	Which phase transitions are not present in the training set?
T7	In which phase are the Scissors used most often?
T8	Which surgeries end in the Cleaning coagulation phase?
T9	Which instrument combination is not present in the training set?
T10	In which phase do Bipolar, Irrigator and SpecimenBag co-occur?

Table 2 shows the dataset split of the Cholec80 dataset that has been used in the user study.

Table 2 Split of the Cholec80 dataset that was used in the user study.

Set	File IDs
Training	1, 2, 3, 4, 5, 6, 7, 8, 9, 11, 12, 14, 15, 16, 18, 20, 21, 24, 25, 26, 27, 28, 30, 31, 34, 35, 39, 40, 42, 43, 44, 45, 46, 47, 48, 49, 50, 51, 52, 53, 54, 55, 56, 58, 59, 61, 62, 63, 65, 67, 68, 69, 71, 72, 73, 75, 76, 77, 80
Validation	17, 36, 37, 41, 57, 60, 64, 66, 70, 74, 78, 79
Test	10, 13, 19, 22, 23, 29, 32, 33, 38

Three statements from the SUS questionnaire that received the most positive rating.

- Q4: I think that I would need the support of a technical person to be able to use this system (mean 1.2)
- Q5: I found the various functions in this system were well integrated (mean 4.5)
- Q6: I thought there was too much inconsistency in this system (mean 1.4)

Conversely, we report three statements that were rated least favorably.

- Q3: I thought the system was easy to use (mean 3.9)
- Q7: I would imagine that most people would learn to use this system very quickly (mean 3.9)
- Q10: I needed to learn a lot of things before I could get going with this system (mean 2.1)

Figure 1 shows the user study results for each task. Overall, the majority of the tasks were completed successfully by $\geq 80\%$ of participants. The tasks T2 and T6 represent exceptions with the overall worst completion rate, solved correctly by 30% and 40% of participants respectively.

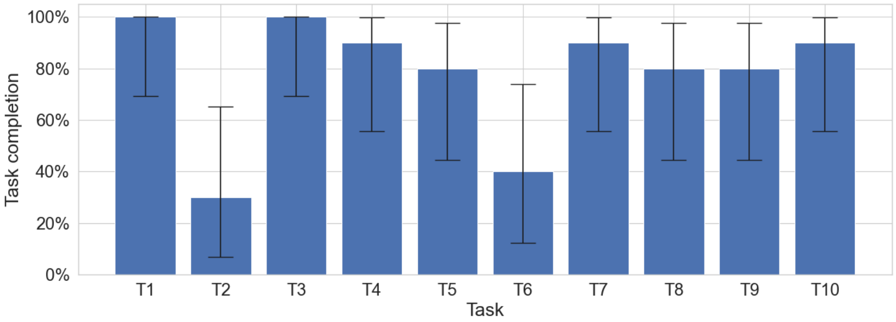

Fig. 1 Overall task completion percentage with the corresponding 95%-confidence intervals.

2 Instrument co-occurrences

The following Tables 3, 4, 5, 6, 7, 8 provide detailed information on instrument co-occurrences in different datasets and splits.

Table 3 Number of frames for each instrument co-occurrence of the Cholec80 dataset with the 40/-/40 split.

Instrument combination	Training	Test
Grasper, Hook	33187	24258
Grasper, Irrigator	2089	1379
Grasper, Bipolar	1552	1857
Grasper, Clipper	2172	1150
Grasper, Scissors	979	681
Grasper, SpecimenBag	3541	4031
Grasper, Bipolar, Irrigator	503	154
Bipolar, Irrigator	229	275
Bipolar, SpecimenBag	127	121
Grasper, Bipolar, SpecimenBag	339	145
Irrigator, SpecimenBag	292	193
Bipolar, Irrigator, SpecimenBag	195	81
Grasper, Irrigator, SpecimenBag	533	242
Grasper, Clipper, Irrigator	12	0
Clipper, Irrigator	4	0
Grasper, Clipper, SpecimenBag	1	0
Grasper, Scissors, SpecimenBag	28	4
Hook, Irrigator	57	474
Grasper, Hook, Irrigator	136	64
Bipolar, Scissors	76	0
Grasper, Hook, SpecimenBag	0	42
Hook, SpecimenBag	0	1
Scissors, Irrigator, SpecimenBag	0	1
Grasper, Scissors, Irrigator	0	2

Table 4 Number of frames for each instrument co-occurrence of the Cholec80 dataset with the 32/8/40 split.

Instrument combination	Training	Validation	Test
Grasper, Hook	26942	6245	24258
Grasper, Irrigator	1665	424	1379
Grasper, Bipolar	1290	262	1857
Grasper, Clipper	1595	577	1150
Grasper, Scissors	788	191	681
Grasper, SpecimenBag	2904	637	4031
Grasper, Bipolar, Irrigator	503	0	154
Bipolar, Irrigator	229	0	275
Bipolar, SpecimenBag	120	7	121
Grasper, Bipolar, SpecimenBag	287	52	145
Irrigator, SpecimenBag	281	11	193
Bipolar, Irrigator, SpecimenBag	195	0	81
Grasper, Irrigator, SpecimenBag	448	85	242
Grasper, Clipper, Irrigator	12	0	0
Clipper, Irrigator	4	0	0
Grasper, Clipper, SpecimenBag	1	0	0
Grasper, Scissors, SpecimenBag	28	0	4
Hook, Irrigator	57	0	474
Grasper, Hook, Irrigator	136	0	64
Bipolar, Scissors	76	0	0
Grasper, Hook, SpecimenBag	0	0	42
Hook, SpecimenBag	0	0	1
Scissors, Irrigator, SpecimenBag	0	0	1
Grasper, Scissors, Irrigator	0	0	2

Table 5 Number of frames for each instrument co-occurrence of the Cholec80 dataset with the 40/8/32 split.

Instrument combination	Training	Validation	Test
Grasper, Hook	33187	5765	18493
Grasper, Irrigator	2089	309	1070
Grasper, Bipolar	1552	415	1442
Grasper, Clipper	2172	321	829
Grasper, Scissors	979	232	449
Grasper, SpecimenBag	3541	965	3066
Grasper, Bipolar, Irrigator	503	47	107
Bipolar, Irrigator	229	66	209
Bipolar, SpecimenBag	127	15	106
Grasper, Bipolar, SpecimenBag	339	9	136
Irrigator, SpecimenBag	292	47	146
Bipolar, Irrigator, SpecimenBag	195	4	77
Grasper, Irrigator, SpecimenBag	533	39	203
Grasper, Clipper, Irrigator	12	0	0
Clipper, Irrigator	4	0	0
Grasper, Clipper, SpecimenBag	1	0	0
Grasper, Scissors, SpecimenBag	28	0	4
Hook, Irrigator	57	474	0
Grasper, Hook, Irrigator	136	64	0
Bipolar, Scissors	76	0	0
Grasper, Hook, SpecimenBag	0	0	42
Hook, SpecimenBag	0	0	1
Scissors, Irrigator, SpecimenBag	0	0	1
Grasper, Scissors, Irrigator	0	0	2

Table 6 Number of frames for each instrument co-occurrence of the CATARACTS dataset with the 25/5/20 split.

Instrument combination	Training	Validation	Test
Bonn forceps, secondary incision knife	38	10	30
phacoemulsifier handpiece, micromanipulator	1977	326	1754
irrigation/aspiration handpiece, micromanipulator	256	69	469
biomarker, Mendez ring	0	1	0
Bonn forceps, primary incision knife	43	11	27
vitrectomy handpiece, micromanipulator	280	0	59
Bonn forceps, implant injector	0	1	3
viscoelastic cannula, micromanipulator	52	0	12
hydrodissection canula, micromanipulator	4	0	3
Bonn forceps, Troutman forceps	0	0	1
Troutman forceps, suture needle	6	8	5
Bonn forceps, Troutman forceps, suture needle	5	6	0
Bonn forceps, suture needle	1	4	0
Bonn forceps, phacoemulsifier handpiece	5	0	13
capsulorhexis forceps, micromanipulator	6	0	23
Bonn forceps, needle holder, suture needle	3	2	2
needle holder, suture needle	5	0	1
capsulorhexis forceps, Vannas scissors	7	9	0
Bonn forceps, capsulorhexis forceps, suture needle	0	0	3
capsulorhexis cystotome, Bonn forceps	57	0	3
Troutman forceps, micromanipulator	2	0	0
capsulorhexis forceps, suture needle	4	0	0

Table 7 Number of frames for each instrument co-occurrence of the CaDIS dataset with the 19/3/3 split.

Instrument combination	Training	Validation	Test
Bonn Forceps, Secondary Knife, Secondary Knife Handle	37	8	5
Secondary Knife, Secondary Knife Handle	69	4	10
Bonn Forceps, Primary Knife	113	9	30
Capsulorhexis Cystotome, Capsulorhexis Cystotome Handle	60	11	13
Phacoemulsifier Handpiece, Phacoemulsifier Handpiece Handle	9	3	0
Phacoemulsifier Handpiece, Micromanipulator, Phacoemulsifier Handpiece Handle	46	4	7
Irrigation/Aspiration Handpiece, Irrigation/Aspiration Handpiece Handle	56	7	21
Lens Injector, Lens Injector Handle	19	13	8
Irrigation/Aspiration Handpiece, Micromanipulator, Irrigation/Aspiration Handpiece Handle	10	0	6
Irrigation/Aspiration Handpiece, Micromanipulator	48	29	10
Rycroft Cannula, Rycroft Cannula Handle	52	18	14
Bonn Forceps, Secondary Knife	75	10	20
Phacoemulsifier Handpiece, Micromanipulator	239	42	44
Primary Knife, Irrigation/Aspiration Handpiece, Micromanipulator	0	0	1
Bonn Forceps, Capsulorhexis Forceps	12	0	9
Irrigation/Aspiration Handpiece, Capsulorhexis Forceps	2	0	0
Micromanipulator, Capsulorhexis Forceps	3	0	0
Bonn Forceps, Phacoemulsifier Handpiece	1	0	2
Bonn Forceps, Phacoemulsifier Handpiece, Phacoemulsifier Handpiece Handle	2	0	0
Hydrosdissection Cannula, Micromanipulator	1	0	0
Capsulorhexis Cystotome, Bonn Forceps	23	0	0
Primary Knife, Lens Injector	2	0	0
Viscoelastic Cannula, Micromanipulator	24	0	0
Viscoelastic Cannula, Capsulorhexis Forceps	12	0	0

Table 8 Number of frames for each instrument co-occurrence of the M2CAI-tool dataset with the 10/-/5 split.

Instrument combination	Training	Test
Grasper, Hook	6279	3814
Grasper, Clipper	275	177
Grasper, Scissors	141	83
Grasper, SpecimenBag	1140	373
Grasper, Bipolar, SpecimenBag	17	47
Grasper, Bipolar	351	193
Grasper, Irrigator	409	63
Grasper, Irrigator, SpecimenBag	126	1
Irrigator, SpecimenBag	26	1
Grasper, Scissors, SpecimenBag	4	0
Grasper, Scissors, Irrigator	2	0
Bipolar, SpecimenBag	12	2
Bipolar, Irrigator, SpecimenBag	4	0
Bipolar, Irrigator	1	28
Grasper, Bipolar, Irrigator	0	8

3 Improved dataset splits

The following Tables 9, 10, 11, 12, 13, 14, 15 define improved dataset splits that are proposed as part of this work.

Table 9 Improved 40/-/40 split of the Cholec80 dataset.

Set	File IDs
Training	1, 2, 3, 4, 5, 6, 7, 8, 9, 10 , 11, 12, 13, 14, 15, 16, 17, 18, 19, 20, 21, 22, 23, 24, 25, 26, 27, 28, 30, 31, 34, 35, 36, 37, 39, 40, 58, 66, 71, 78
Test	29, 32, 33, 38, 41, 42, 43, 44, 45, 46, 47, 48, 49, 50, 51, 52, 53, 54, 55, 56, 57, 59, 60, 61, 62, 63, 64, 65, 67, 68, 69, 70, 72, 73, 74, 75, 76, 77, 79, 80

Table 10 Improved 32/8/40 split of the Cholec80 dataset.

Set	File IDs
Training	1, 2, 3, 4, 5, 6, 7, 8, 9, 10, 11, 12, 13, 15, 16, 17, 18, 19, 20, 21, 22, 24, 25, 26, 27, 28, 30, 31, 37, 41, 57, 60
Validation	14, 33, 34, 35, 36, 38, 39, 40
Test	23, 29, 32, 42, 43, 44, 45, 46, 47, 48, 49, 50, 51, 52, 53, 54, 55, 56, 58, 59, 61, 62, 63, 64, 65, 66, 67, 68, 69, 70, 71, 72, 73, 74, 75, 76, 77, 78, 79, 80

Table 11 Improved 40/8/32 split of the Cholec80 dataset.

Set	File IDs
Training	1, 2, 3, 4, 5, 6, 7, 8, 9, 10, 11, 12, 13, 15, 16, 17, 18, 19, 20, 21, 22, 24, 25, 26, 27, 28, 30, 31, 34, 35, 36, 37, 39, 40, 43, 46, 47, 48, 60, 70
Validation	14, 33, 38, 41, 42, 44, 45, 57
Test	23, 29, 32, 49, 50, 51, 52, 53, 54, 55, 56, 58, 59, 61, 62, 63, 64, 65, 66, 67, 68, 69, 71, 72, 73, 74, 75, 76, 77, 78, 79, 80

Table 12 Improved 25/5/20 split of the CATARACTS dataset.

Set	File IDs
Training	14, 26, 27, 28, 29, 30, 31, 32, 33, 34, 36, 37, 38, 39, 40, 41, 42, 43, 44, 45, 46, 47, 48, 49, 50
Validation	1, 7, 16, 19, 35
Test	2, 3, 4, 5, 6, 8, 9, 10, 11, 12, 13, 15, 17, 18, 20, 21, 22, 23, 24, 25

Table 13 Improved 19/3/3 split of the CaDIS dataset.

Set	File IDs
Training	1, 2, 3, 4, 6, 7, 8, 9, 10, 11, 13, 14, 15, 17, 18, 20, 23, 24, 25
Validation	5, 16, 19
Test	12, 21, 22

Table 14 Improved 27/-/14 split of the M2CAI-workflow dataset.

Set	File IDs
Training	train1, train2, train3, train4, train5, train6, train7, train8, train9, train11, train12, train13, train14, train15, train16, train17, train18, train19, train20, train21, train22, train23, train24, train25, train26, train27, test11
Test	train10, test1, test2, test3, test4, test5, test6, test7, test8, test9, test10, test12, test13, test14

Table 15 Improved 10/-/5 split of the M2CAI-tool dataset.

Set	File IDs
Training	1, 2, 3, 4, 5, 7, 8, 9, 10, 14
Test	6, 11, 12, 13, 15
